# Analysis of spatial patterns and influencing factors of farmland transfer in China based on ESDA-GeoDetector

**DOI:** 10.1038/s41598-024-62931-1

**Published:** 2024-05-31

**Authors:** Xiuli He, Wenxin Liu

**Affiliations:** 1 Northeast Institute of Geography and Agroecology, Chinese Academy of Sciences, Changchun, 130102 China; 2 College of Resources and Environment, University of Chinese Academy of Sciences, Beijing, 101314 China

**Keywords:** Ecology, Environmental sciences

## Abstract

Farmland transfer is a critical component in facilitating agricultural scale management and improving agricultural production efficiency. This study examines the spatial distribution of farmland transfer in China and identifies the factors influencing it, offering valuable guidance for advancing China’s farmland transfer practices. Through the application of mathematical statistics and GIS spatial analysis, the study investigates changes in spatial patterns related to the scale, rate, mode, and recipients of farmland transfer across China's 30 provinces from 2015 to 2020. Geographical detectors are also employed to identify the key factors influencing the extent and pace of farmland transfer. The study reveals that between 2015 and 2020, China's farmland transfer area increased from 29,789 to 37,638 million hectares. Provinces with abundant farmland resources generally experienced larger farmland transfers, while economically developed regions and major grain-producing areas saw higher rates of farmland transfers. The predominant mode of farmland transfer in China was leasing (subcontracting), accounting for over 80% of the total transferred area. Large-scale grain growers and family farms were significant participants in farmland transfers, acquiring approximately 60.1% of the transferred lands, followed by professional cooperatives (21.5%), enterprises (10.4%), and other entities (7.9%). Key factors influencing the farmland transfer area include the "regional farmland area", the "proportion of family farms supported by loans", and the "proportion of non-agricultural population", with explanatory powers of 0.663, 0.319, and 0.225, respectively. Notably, there is a substantial interaction between the "regional farmland area" and factors such as the "proportion of family farms supported by loans" and the "grain yield per unit area", with explanatory powers reaching 0.957 and 0.901, respectively. These findings offer valuable insights for promoting farmland transfer in agriculturally rich regions. Factors affecting the farmland transfer rate include "grain yield per unit area", "GDP per capita", and the "proportion of non-agricultural population", each with an explanatory power above 0.500. Moreover, their interactive explanatory powers with other indicators exceed 0.600, indicating that provinces with high agricultural productivity or economic development levels are more likely to undergo farmland transfer. The paper concludes by proposing strategies and recommendations to promote farmland transfer in both "large agricultural areas" and "metropolitan suburbs."

## Introduction

Food security serves as a crucial cornerstone for national security. Facilitating farmland transfer and expanding the scale of peasant household operations represent significant approaches for China to enhance grain production efficiency and ensure national food security. With over 230 million small farmers in China, each possessing an average farmland area of less than 0.5 hectares per household, the limited scale of agricultural operations has somewhat impeded the development of a modern agricultural economy. For more than a decade, the Chinese government has been actively promoting farmland transfer and appropriate scale management within agriculture as a pivotal agricultural policy measure. In 2007, the Property Law of the People's Republic of China authorized the transfer of contracted land management rights. The "No. 1 central document" from 2008 to 2013 emphasized the necessity of promoting moderate-scale operations and cultivating new agricultural operators. A series of proactive and explicit policies have expedited farmland transfer in China^1^.

In the relevant research of countries with private land ownership, the focus is on the transaction of land ownership and its impact on the intensive use of farmland^2^. Many scholars argue that establishing a well-defined system of property rights is crucial for facilitating the transfer of farmland, thereby enabling large-scale management, and enhancing agricultural production efficiency throughout the process^3^. Effective land transfer policies and standardized land transfer markets are essential for gradually transferring farmland to more efficient operators. The high cost of farmland transfer, however, has been found in some studies to diminish operators' willingness to transfer and exert a noticeable inhibitory effect on the transfer of farmland^4,5^. Furthermore, the influence of government intervention on farmland transfer is also an important research area. Some scholars argue that excessive government intervention can hinder the development and utilization of farmland resources, thereby significantly reducing the efficiency of farmland transfer^6^. However, studies conducted by other scholars have demonstrated that government policies aimed at promoting social and economic development and addressing market failures have effectively mitigated the impact of information asymmetry on the transfer of farmland, thereby playing a constructive role in facilitating such transfers^7^.

China's rural land system operates under collective ownership, and the transfer of rural land began in the 1980s. By 2000, only about 3% of China's farmland was involved in such transfers. Subsequently, with the implementation of a series of relevant laws and regulations such as the "Land Contract Law", there has been a significant increase in China's farmland transfer. In recent years, the proportion of transferred farmland area relative to China's total farmland reached about 37%, but it has slightly decreased to around 36% at present. From a research perspective, existing studies predominantly support farmland transfer and acknowledge its contribution to the development of agriculture, rural areas, and farmers. For instance, villages with high rates of farmland turnover tend to have relatively low abandonment rates^8^. Furthermore, transferring farmland can enhance crop production efficiency and effectively reduce income disparities in rural regions^9–11^. Moreover, the scope of research has gradually expanded from investigating the scale of farmland transfer to comprehensively analyzing its modes, objects, and influencing factors. Reviewing current research findings reveals that studies on influencing factors primarily focus on three main aspects^12–15^.

(1) Policy factors. Apart from the impact of land policies, various agricultural subsidy policies also significantly influence farmland transfer in China. Some studies indicate that innovations in the land ownership system do not have a substantial effect on agricultural land transfer. However, the confirmation, registration, and certification of rural contracted land significantly promote farmers' willingness to transfer farmland but do not significantly affect their desire to transfer it^16^. Direct grain subsidies and subsidies for improved varieties encourage the outflow of farmland, whereas comprehensive subsidies for agricultural materials and subsidies for purchasing agricultural machinery inhibit such outflow^17–19^. Grain price support policies have a significant impact on the outflow of farmland. Although rising grain prices may drive up the cost of land transfers, they also play a crucial role in ensuring the income of large-scale operations from grains^15^. (2) Regional characteristics. In the case study of the entire country, the conversion of farmland is influenced by various factors, including the economy, income, and agricultural mechanization. Among these factors, agricultural mechanization stands out as the most significant one. When examining the results of farmland transfers at a township level, it becomes evident that small-scale transfers are primarily affected by the number of agricultural labor force and the commercialization rate of aquaculture industry. The former shows a negative correlation, while the latter exhibits a positive correlation with such transfers^13^. Furthermore, different agricultural areas are subject to various factors that influence their land transfer dynamics. In rural mountainous regions with limited resources, per capita household income and labor force play crucial roles in determining land transfer patterns^20^. On the other hand, major grain-producing areas experience influences from education level, income source diversification, new agricultural management subjects as well as market conditions for land transfer. Additionally to labor force availability, mechanization levels and income levels also impact land transfers in agriculture and animal husbandry areas where animal husbandry income proportion plays an important role too^21–23^. Moreover, in northern mountainous regions there is a decrease in farmland transfer rates with increasing elevation; villages with average farmland slopes of 6° or less exhibit relatively higher rates^24^. (3) Farmer attributes. The study of farmers' characteristics in farmland transfer primarily relies on questionnaires^25^. Due to the categorization of farmers into pure farmers and part-time farmers, their motivations and behaviors towards land transfer differ significantly^26,27^. Pure farmers prioritize agricultural production, thus their decision to transfer farmland is mainly influenced by survival factors and economic considerations^28^. When agricultural profits are low, pure farmers may be more inclined to transfer their land; conversely, as agricultural incomes increase, their willingness to do so declines. On the other hand, for part-time farmers^29,30^, the level of regional economic development plays a crucial role in determining whether they choose to transfer farmland. The availability of non-agricultural employment opportunities makes it easier for part-time farmers to engage in such transfers^26–28^.

In recent years, the Chinese government has implemented strict regulations to prohibit the occurrence of "non-grain conversion" and "non-agricultural conversion" during the process of farmland transfer. As a result, most of the transferred farmland has not undergone significant changes in land use, with agricultural cultivation remaining as the primary purpose after transfer. Consequently, there has been minimal alteration in income per unit of farmland area. Based on data regarding farmland transfer, China's annual transfer area has remained relatively stable at around 35 million hectares since 2015, indicating that this amount is susceptible to outflow. However, small-scale farmers who continue to operate their own farmland exhibit a strong attachment to it due to limited alternative employment opportunities. Considering factors such as current economic conditions, policy provisions, market environment, circulation income and others, entities opting for scale management within agriculture have also reached a certain level of equilibrium or saturation unless more favorable conditions emerge to break this impasse. The current research results focus on two aspects: one is to analyze the extent to which the transfer of farmland benefits agriculture, rural areas, and farmers; the other is to delve into the constraining factors of farmland transfer from various perspectives, such as systemic factors, subsidies, and farmers' willingness. Existing findings can confirm that farmland transfer plays a positive role in promoting the development of agriculture, rural areas, and farmers. However, when relying solely on a single perspective or micro-investigation approach, it becomes challenging to elucidate the driving forces or constraints behind farmland transfer at a macro level. During the period of rapid growth in farmland transfer, individual factors can effectively facilitate this process. Currently, the slow growth in farmland transfer rates necessitates a systematic consideration of regional economic development levels, agricultural operational conditions, farmland output capacity, policies, market dynamics, and farmers' attributes to promote this practice^31^. Only through such comprehensive analysis can an appropriate strategy for farmland transfers be formulated based on local circumstances^32^. This study adopts 30 provinces in China as the research unit (excluding Tibet, Hong Kong, Macao, and Taiwan) to comprehensively assess the temporal and spatial characteristics of farmland transfer in China. It considers a range of internal and external factors, such as regional economic development level, urbanization level, resource endowment conditions, agricultural production level, and policy subsidies, to identify the key determinants influencing the extent and rate of arable land transfer. The aim is to provide valuable insights for the sustainable development of farmland transfer in China.

## Methods and data

### Analytical framework

**Figure 1:**
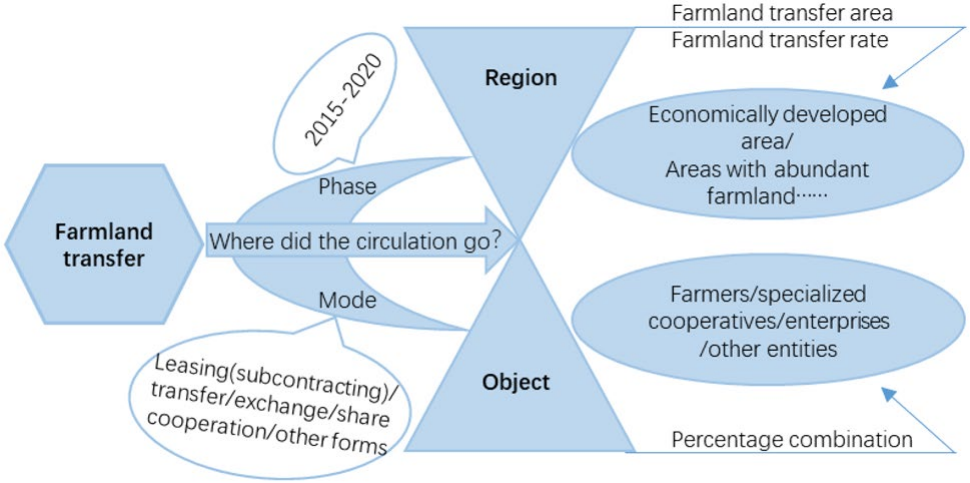
Research diagram of the spatial–temporal pattern of farmland transfer in China.

To analyze the process of China’s farmland transfer comprehensively, it is imperative to address the following questions for a more thorough exploration of the primary factors influencing farmland transfer: at what stage does the transfer of farmland occur? What are the main regions where farmland is distributed? In what mode is farmland transferred? To whom is farmland transferred? To provide a clearer illustration of these issues, refer to Fig. 1.

### Indicators selection

In this paper, the influencing factors of farmland transfer are analyzed using a geographic detector method, and a comprehensive index system is established during the calculation process. Among them, two dependent variables are set: the farmland transfer area (Y1) and the farmland transfer rate (Y2). This is because there are significant regional differences in both the size and rate of farmland transfer among provinces. For instance, in 2020, the range of farmland transfer areas across provinces varied from 112,000 hectares to 7.578 million hectares. Similarly, the proportion of farmland transfer areas ranged from 9.2 to 91.1%, with cases where there was a low farmland transfer area but a high farmland transfer rate or vice versa. Therefore, we have respectively designated the farmland transfer area and rate as dependent variables for two separate analyses to more accurately examine influencing factors associated with different types of farmland transfers. The 2 dependent variables and 10 independent variables involved in the study are listed in Table 1 across 6 facets. (1) Level of economic development. The indicator used to measure this is "GDP per capita" (X1). A higher level of economic development typically attracts a larger labor force from the primary industry to the secondary and tertiary industries, thereby facilitating the transition of farmland. (2) Agricultural operation conditions. The indicators include "regional farmland area" (X2), "total power of agricultural machinery per hectare" (X3), and "proportion of disaster-affected area" (X4). The first two indicators positively influence the farmland transfer, representing the availability of farmland for transfer and the level of modernization in agricultural production. Conversely, the proportion of affected area negatively correlates with farmland transfer. The more natural disturbances affect farmland output, the more challenging it becomes to facilitate its transfer. (3) Farmland output capacity. The characteristic index is "grain yield per unit area" (X5), which is influenced by both natural and human factors. It reflects natural factors such as soil texture, organic matter content, surface thickness, soil pH value, and slope elevation to a certain extent. Selecting this index can effectively reduce unnecessary duplication among certain indicators^33^. (4) Scale operational conditions. The indicators encompass the "proportion of farmland transferred through leasing (subcontracting)" (X6) and the "proportion of farmland transferred to family farms and cooperatives" (X7). The former measures the extent of cultivation in farmland transfer channels, while the latter assesses the capacity of new agricultural production and management entities to undertake farmland. (5) The intensity of policy support. A representative index is the "proportion of family farms supported by loans" (X8). The process of large-scale farmland operation in China is closely intertwined with policy orientation. Financial support significantly influences both the scale and speed at which farmland transfers occur. (6) Farmers' off-farm attributes. Representational indicators include the "proportion of non-agricultural population" (X9) and the "proportion of migrant labor force" (X10). The more individuals who are rooted in rural areas, the higher their difficulty coefficient in transferring farmland becomes; conversely, a lower proportion eases this coefficient.

**Table 1:** Variables and their descriptive statistics (2020 data).

Indicators	Units	Serial number	Minimum	Maximum	Mean	Standard deviation
Farmland transfer area	Ten thousand hectares	Y1	3.96	438.70	125.46	100.26
Farmland transfer rate	%	Y2	9.19	91.11	38.47	17.94
GDP per capita	Yuan	X1	35,995.00	164,889.00	71,401.30	31,138.20
Regional farmland area	Ten thousand hectares	X2	11.19	757.81	347.04	226.62
Total power of agricultural machinery per hectare	Kw/ha	X3	3.99	12.24	6.56	2.08
Proportion of disaster-affected area	%	X4	1.63	30.82	10.71	8.42
Grain yield per unit area	kg/ha	X5	3703.45	7996.50	5705.18	969.72
Proportion of farmland transferred by leasing (subcontracting)	%	X6	66.72	98.59	84.09	8.83
Proportion of farmland transferred to family farms and cooperatives	%	X7	5.25	68.10	33.06	12.82
Proportion of family farms supported by loans	%	X8	0.14	10.48	2.13	2.20
Proportion of non-agricultural population	%	X9	50.05	89.30	64.66	9.76
Proportion of migrant labor force	%	X10	26.91	57.39	44.18	8.56

According to the geographical detector method, the variables were classified into type quantity using the natural break method. The negative correlation indicator, "proportion of disaster-affected area", was assigned values 1, 2, 3, 4, and 5 based on its descending value. The other positive correlation indicators were assigned values 5, 4, 3, 2 and 1 in descending order according to their respective values.

## Research methods

### Global spatial autocorrelation

The concept of global spatial autocorrelation refers to the characterization of the spatial attributes of geographic elements across an entire region^34^. The formula for calculating the global Moran's index is as follows:

$$I=\frac{n\sum_{i=1}^{n}{\sum }_{j=1}^{n}{w}_{ij}({x}_{i}-\overline{x })({x}_{j}-\overline{x })}{\sum_{i=1}^{n}\sum_{j=1}^{n}{w}_{ij}{({x}_{i}-\overline{x })}^{2}}$$

where, *I* is the global Moran’ index; *n* is the number of evaluation units; and ${x}_{i}$, ${x}_{j}$ $\text{is}$ the farmland transfer rate of province *i* and province *j*; $\overline{x }$ is the average level of farmland transfer rate of all provinces; ${w}_{ij}$ is the aggregation of all spatial weights.

The value of *I* ranges from − 1 to 1, with positive values indicating positive correlation in spatial distribution of factor attribute values, negative values indicating negative correlation, and zero indicating no correlation.

### Geographical detectors

The Geographical Detector, developed by Wang Jinfeng, is a specialized tool for detecting and utilizing spatial differentiation. It comprises four detectors: differentiation and factor detection, interaction detection, risk area detection, and ecological detection^35^. This paper focuses on the application of the first two types of detectors.

(1) Differentiation and factor detection primarily investigate the spatial differentiation of the dependent variable Y and the extent to which factor X contributes to the spatial differentiation of Y. This is quantified by the q value, as expressed below:

$$\text{q}=1-\frac{\sum_{h=1}^{L}{N}_{h}{\sigma }_{h}^{2}}{N{\sigma }^{2}}=1-\frac{SSW}{SST}$$

$$\text{SSW}=\sum_{h=1}^{L}{N}_{h}{\sigma }_{h}^{2}$$

$$\text{SST}=\text{N}{\sigma }^{2}$$

where, *h* is the layering of dependent variable Y or factor X, ${N}_{h}$ and N is the number of units in layer *h* and the whole region, and ${\sigma }_{h}^{2}$ and ${\sigma }^{2}$ is the variance of Y values in layer *h* and the whole region. SSW and SST are the sum of variances within layers and the total variances of the whole region. The range of q is [0, 1], and the larger the value, the more obvious the spatial differentiation of Y. q = 0 indicates that X has no relationship with Y, and q = 1 indicates that X completely controls the spatial distribution of Y. The larger the value of q indicates that X has stronger ability to explain Y, and vice versa. The value of q reflects that X explains 100*q% of Y.

(2) Interaction detection is utilized to identify the interplay among various influencing factors and assess the explanatory capacity of their combined impact on the dependent variable Y. Firstly, the q values of the two factors X1 and X2 for Y, q(X1) and q(X2), are calculated respectively, and then the q values for their interaction, q(X1 ∩ X2), are calculated. Finally, q(X1), q(X2) are compared with q(X1 ∩ X2). Table 2 shows the types of interactions between these two factors.

**Table 2:** Types of two-factor interactions.

Criteria	Interaction
q(X1 ∩ X2) < Min(q(X1), q(X2))	Nonlinearity attenuation
Min(q(X1) < q(X2) < q(X1 ∩ X2) < Max(q(X1), q(X2))	Single-factor nonlinearity diminishes
q(X1 ∩ X2) > Max(q(X1), q(X2))	Two-factor enhancement
q(X1 ∩ X2) = (q(X1) + q(X2)	Independent
q(X1 ∩ X2) > (q(X1) + q(X2)	Nonlinear enhancement

### Percentage combination method

When describing the mode and object of farmland transfer, the percentage combination is used for determination. For example, if mode A represents more than 80% of the total area of farmland transfer, then the region's farmland transfer mode is classified as A. If the proportion of farmland transfer area for any individual mode falls below 80%, but the combined ratio of modes A and B exceeds 80%, then the farmland transfer mode in this region is categorized as A + B. Similarly, if A + B is less than 80%, but A + B + C exceeds 80%, then the farmland transfer mode in this area would be identified as A + B + C.

### Data sources and *indicator* interpretation

The data primarily originates from the China Rural Operation and Management Statistical Annual Report 2015–2018, China Rural Policy and Reform Statistical Annual Report 2019–2020, and China Statistical Yearbook 2016–2021. Indicators related to farmland transfer are sourced from the China Rural Operation and Management Statistical Annual Report, while other data is obtained from the China Statistical Yearbook. Considering the data availability, this paper's analysis encompasses 30 provinces (municipalities and autonomous regions), excluding Tibet, Hong Kong, Macao, and Taiwan. In this study, the farmland transfer area refers to the total contracted area of farmland voluntarily transferred by households to other operators through legal means such as leasing (subcontracting), transfer, exchange, share cooperation, or any other method with compensation.

## Results

### Characteristics of farmland transfer stage

**Figure 2:**
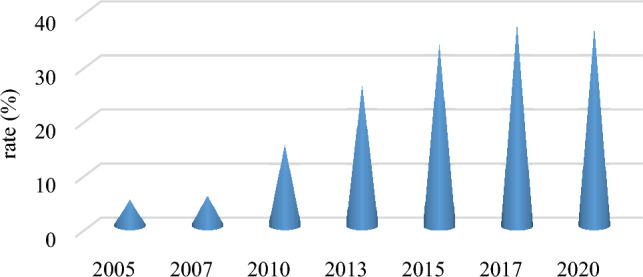
Farmland transfer rate in China from 2005 to 2020.

China's farmland transfer has transitioned from a period of rapid growth to one of slow growth. In the early twenty-first century, China proposed suggestions to promote farmland transfer and gradually standardized such practices. As depicted in Fig. 2, the area of farmland in China was 3,644,900 hectares in 2005. By 2020, this area had increased more than tenfold to reach 37,637,800 hectares, accounting for an increased proportion of the country's total farmland area from 4.57 to 36.15%. Analyzing different time periods for farmland transfers reveals that during the years between 2005 and 2010 marked the initial phase with an average annual increase of less than two million hectares. From 2010 to 2015, there was a rapid period characterized by farmland transfers, with an average annual increase approaching four million hectares. Between 2015 and 2020, there was a deceleration in farmland transfers, with an average annual increase of around one million hectares. From 2015 to 2020, China's farmland transfer area showed sluggish growth, even lagging the growth rate of total farmland. The proportion of farmland transfer area in the total farmland has shown a downward trend. This phenomenon is closely linked to the frequent implementation of various national policies, such as adjustments in planting structure, temporary purchase and storage prices for corn and soybeans, and subsidies for producer income during this period. It also indicates that approximately one-third of China's farmland is relatively easy to transfer, while the remaining two-thirds face increasingly complex constraints. Therefore, this paper will focus on analyzing data from 2015 to 2020 with the aim of exploring the characteristics of farmland transfer during this period of slow growth and identifying strategies to promote it.

### Spatial pattern of farmland transfer

**Figure 3:**
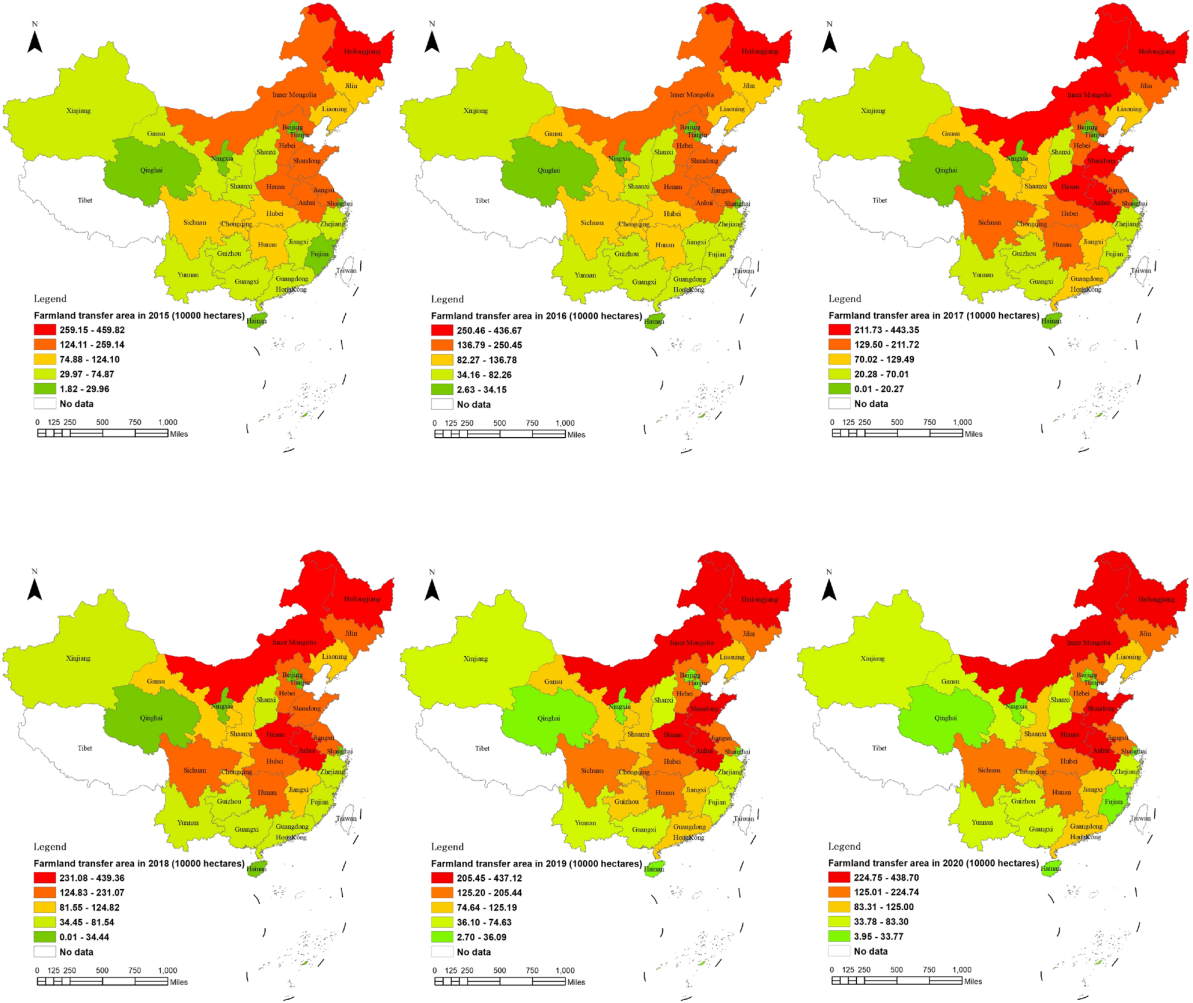
Spatial pattern changes of farmland transfer area in China from 2015 to 2020. *Note* This figure is based on the standard map production of the National Platform for Common GeoSpatial Information Services (http://www.tianditu.gov.cn), with the map approval number of GS(2024)0650, and the base map has not been modified. The software version used for map production is ArcGIS 10.3. URL link: https://www.esri.com/en-us/home.

From 2015 to 2020, the farmland transfer area increased from 29,789 to 37,638 million hectares, and provinces with abundant farmland resources typically experienced significant farmland transfer activity. As depicted in Fig. 3, during this period, the spatial arrangement of the lower grades remained relatively unchanged within the five grades categorized by farmland circulation area. However, a significant number of provinces transitioned from the middle grade to the high grade, resulting in a substantial increase in the number of provinces classified as high-grade. By 2020, Heilongjiang, Shandong, Anhui, Inner Mongolia, Henan, Jiangsu, Jilin, Sichuan, Hebei, Hunan, Hubei, and Liaoning had more than 1.25 million hectares of farmland under circulation. It is worth noting that although China's farmland transfer area continues to expand, its growth rate has decelerated. Notably different from other provinces experiencing continuous growth in this aspect are Heilongjiang and Henan, which witnessed a decrease of 211 thousand hectares and 58 thousand hectares, respectively. This divergence may be one of the key factors contributing to China's slowdown in farmland transfer.

**Figure 4:**
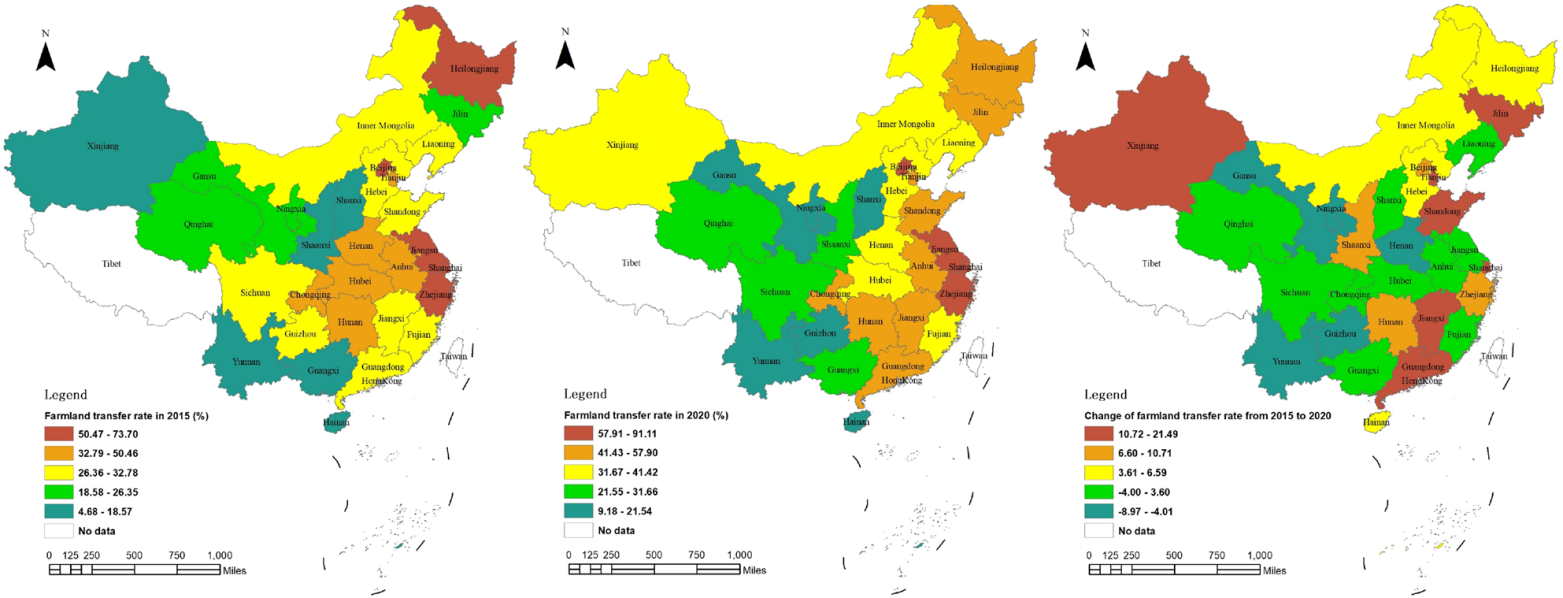
Spatial pattern changes of farmland transfer rate in China from 2015 to 2020. *Note* This figure is based on the standard map production of the National Platform for Common GeoSpatial Information Services (http://www.tianditu.gov.cn), with the map approval number of GS(2024)0650, and the base map has not been modified. The software version used for map production is ArcGIS 10.3. URL link: https://www.esri.com/en-us/home.

From 2015 to 2020, China witnessed an increase in the farmland transfer rate from 33.3 to 36.2%, with approximately 80% of provinces exhibiting an upward trend. Figure 4 illustrates that Shanghai, Beijing, Jiangsu, Zhejiang, Heilongjiang, Guangdong, Anhui, Tianjin, Jiangxi, Hunan, Shandong, Chongqing, and Jilin have higher farmland transfer rates. Notably, Shanghai has reached a remarkable level of 91.1%. When comparing the data between the periods of 2015 and 2020 for farmland transfer rates in various provinces across China, Jilin, Tianjin, Shanghai, Xinjiang, Shandong, Jiangxi, and Guangdong experienced the most significant increases, ranging from approximately15.6 to 21.5 percentage points, respectively. However, Hubei, Gansu, Henan, Ningxia, Yunnan, and Guizhou have consistently maintained low levels of farmland conversion.

**Figure 5:**
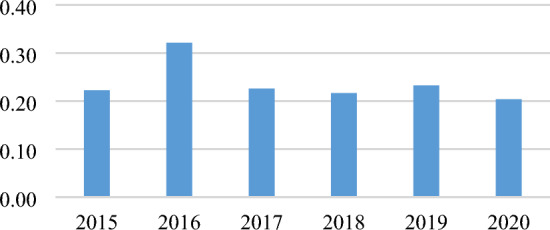
Global Moran’s *I* of farmland transfer rate from 2015 to 2020.

Based on the analysis of spatial autocorrelation, the Global Moran's *I* of farmland transfer rate increased from 0.2226 in 2015 to 0.3214 in 2016 and then decreased to 0.2038 in 2020. This indicates a slight dispersion in the recent years' spatial agglomeration trend. However, as depicted in Fig. 5, the Global Moran's index at the aggregate level exceeded 0.20 and passed the significance test, revealing a significant positive spatial correlation among the farmland transfer rates across China's 30 provinces. As shown in Fig. 4, from 2015 to 2020, the farmland transfer rates in Shanghai, Jiangsu, and Zhejiang have consistently exceeded the national average. These three provinces are spatially adjacent, all situated in the eastern part of China. It is worth noting that the Global Moran's *I* value suddenly increased to 0.3214 in 2016. By analyzing the farmland transfer rates of 30 provinces in 2016, it was found that the farmland transfer rate of Anhui Province, which is adjacent to Zhejiang and Jiangsu, was also higher than the national average and passed the significance test. This increase in the number of neighboring provinces to 4 led to an increase in the Global Moran's *I*.

### Mode of farmland transfer

**Figure 6:**
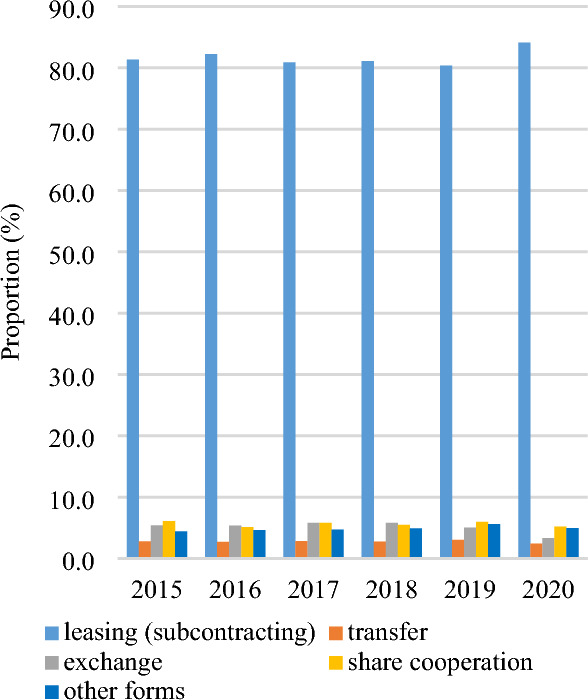
Composition of farmland transfer form in China from 2015 to 2020.

**Figure 7:**
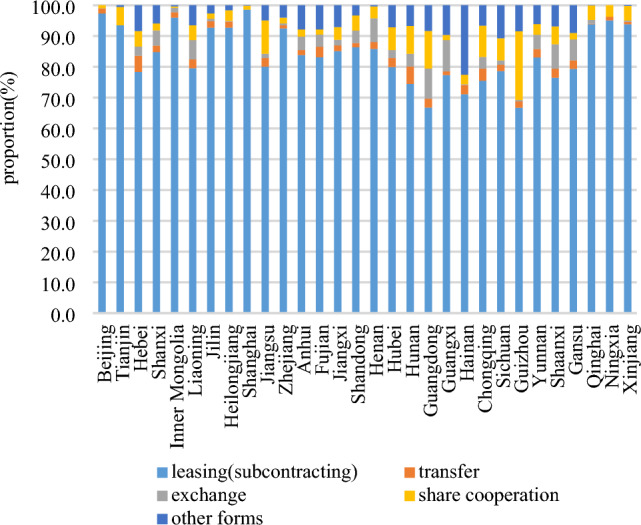
Composition of farmland transfer forms by provincial unit in China in 2020.

**Figure 8:**
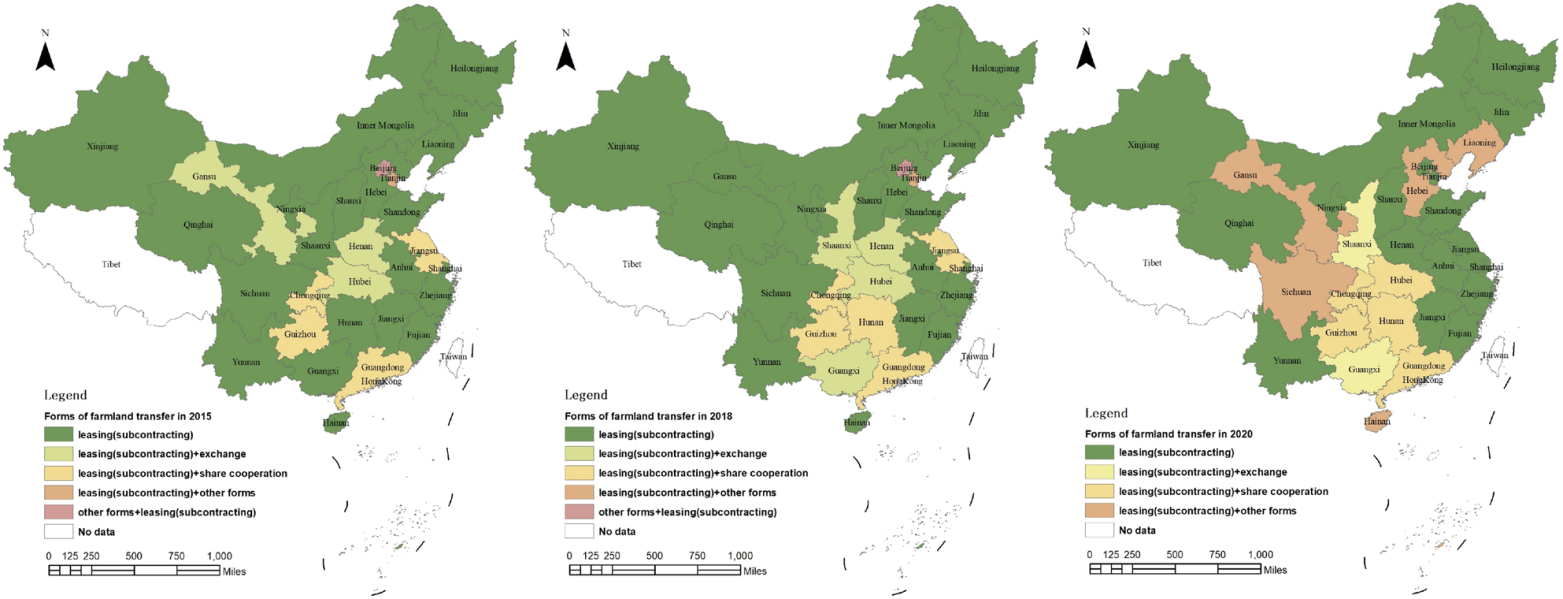
Changes of farmland transfer modes in China from 2015 to 2020. *Note* This figure is based on the standard map production of the National Platform for Common GeoSpatial Information Services (http://www.tianditu.gov.cn), with the map approval number of GS(2024)0650, and the base map has not been modified. The software version used for map production is ArcGIS 10.3. URL link: https://www.esri.com/en-us/home.

Leasing (subcontracting) is the predominant mode of farmland transfer in China. As depicted in Fig. 6, from 2015 to 2020, the proportion of farmland area transferred through leasing (subcontracting) consistently exceeded 80%, indicating a continuous upward trend, while the proportions attributed to transfer, exchange, and share cooperation experienced a decline. In 2020, leasing (subcontracting) accounted for 84.1% of total farmland transfers, while transfer, exchange, share cooperation, and other forms constituted shares of 2.4%, 3.3%, 5.2%, and 5.0%, respectively. The proportion of various farmland transfer modes within each province is illustrated in Fig. 7. Notably, leasing (subcontracting) accounted for over 95% of the farmland transfers in Shanghai, Beijing, Inner Mongolia, and Ningxia. Conversely, the highest proportions of transfers through methods such as transfer, exchange, share cooperation, and other forms were observed in Hunan, Guangxi, Guizhou, and Hainan at rates of 5.6%, 10.2%, 22.3%, and 22.5%, respectively. The spatial pattern of farmland transfer modes in provincial units is depicted in Fig. 8. In 2015, China had five types of farmland transfer modes: leasing (subcontracting), leasing (subcontract) + exchange, leasing (subcontract) + share cooperation, leasing (subcontract) + other forms, and other forms + leasing (subcontract). By 2020, the number of categories had been reduced to four, with no provincial units utilizing "other forms + leasing (subcontracting)" as the primary mode of transfer. Notably, areas where "leasing (subcontracting) + " serves as the main mode have transitioned from being scattered to contiguous. In most major grain-producing regions, over 80% of farmland transfers occur through leasing (subcontracting).

### Object of farmland transfer

**Figure 9:**
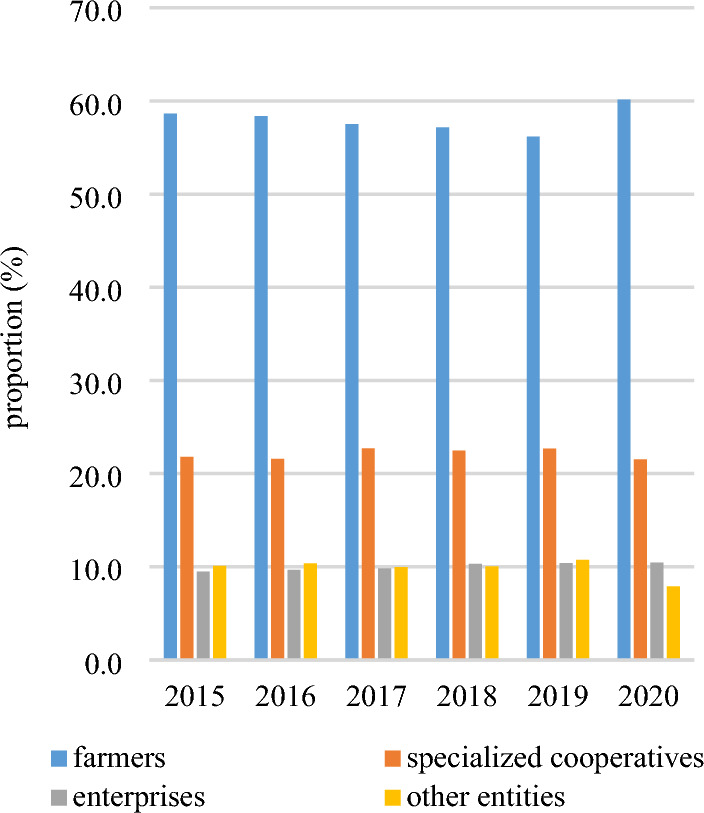
Proportion of farmland flowing into various objects in China from 2015 to 2020.

**Figure 10:**
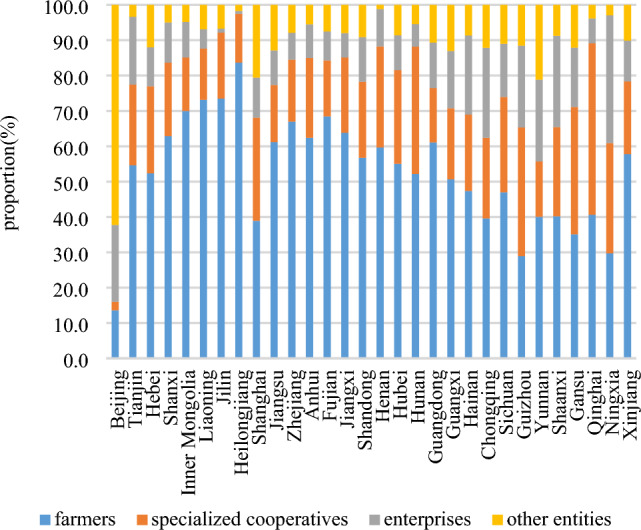
Composition of farmland transfer and destination of each provincial unit in China in 2020.

**Figure 11:**
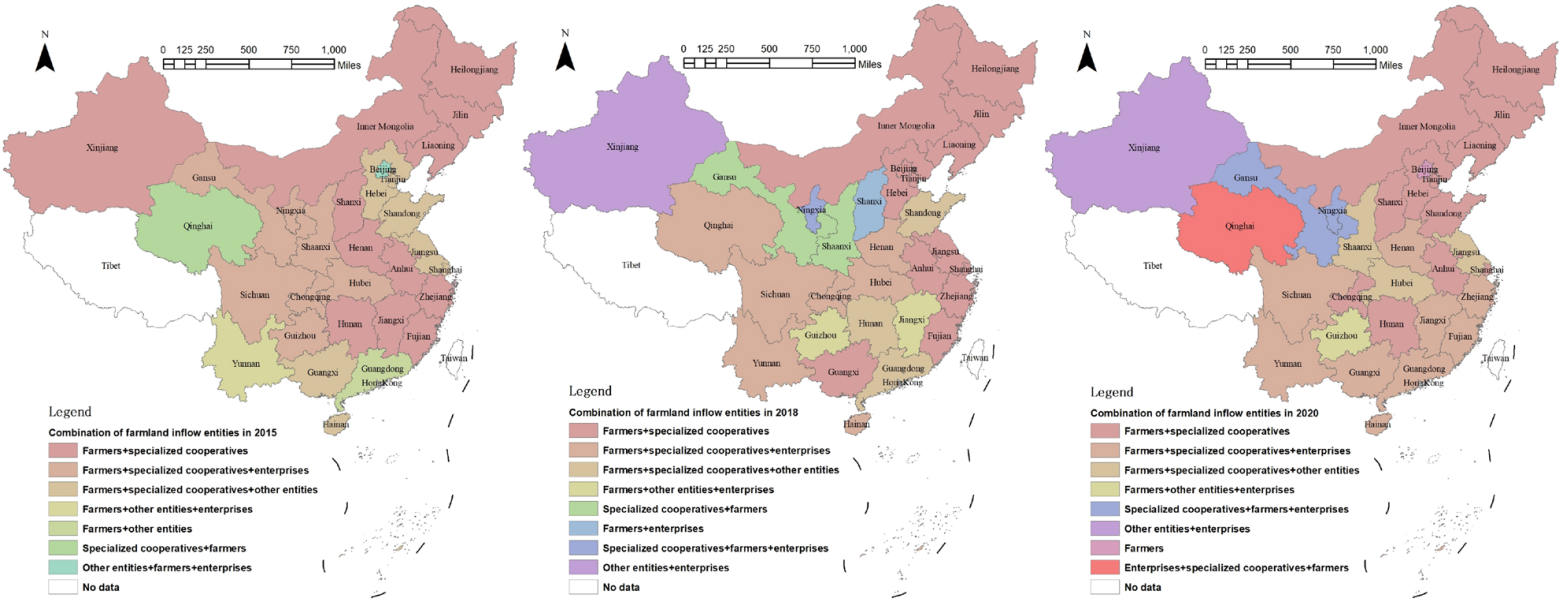
Comparison of the direction of farmland transfer in China from 2015 to 2020. *Note* This figure is based on the standard map production of the National Platform for Common GeoSpatial Information Services (http://www.tianditu.gov.cn), with the map approval number of GS(2024)0650, and the base map has not been modified. The software version used for map production is ArcGIS 10.3. URL link: https://www.esri.com/en-us/home.

The primary object of farmland transfer is farmers, encompassing both large-scale grain growers and family farms. As depicted in Fig. 9, the distribution ratio of farmland transfer among different objects remains relatively stable from 2015 to 2020. Notably, there has been a slight increase in the proportion of farmland transferred to farmers and enterprises, while a minor decrease can be observed in the proportion transferred to specialized cooperatives and other entities. Overall, the primary object of farmland transfer are generally farmers, while specialized cooperatives serve as the secondary object. In 2020, approximately 60.1% of farmland was transferred to farmers, while specialized cooperatives, enterprises, and other entities will receive shares amounting to 21.5%, 10.4%, and 7.9% respectively. It is worth noting that in Fig. 10, 48.5% of farmland was transferred to specialized cooperatives in Qinghai, while 36.1% was transferred to enterprises in Ningxia and 62.3% was transferred to other entities in Beijing. This data highlights the significant role played by specialized cooperatives, enterprises, and other entities as important recipients of farmland transfers. Figure 11 illustrates the spatial transformations of farmland transfer objects in each provincial unit. The period from 2015 to 2020 witnessed the existence of approximately 7–8 types of farmland transfer objects and their various combinations in China. While the number remains relatively stable, the combination forms undergo significant changes. Apart from the enduring combinations such as farmers + specialized cooperatives, farmers + specialized cooperatives + enterprises, farmers + specialized cooperatives + other subjects, and farmers + other subjects + enterprises across different periods, certain combinations of circulation objects have vanished while new ones have emerged. In 2010, a new type of combination involving individual farmers, enterprises and professional cooperatives was formed, which was unprecedented.

### Influencing factors of farmland transfer

#### Key factors influencing farmland transfer area

**Figure 12:**
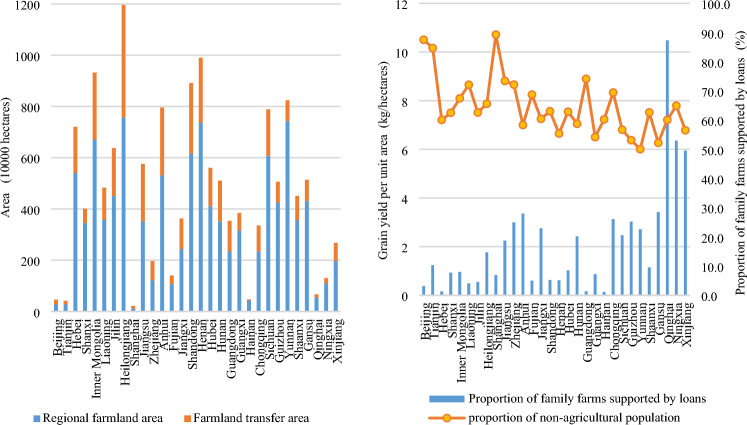
The comparative analysis of evaluation indicators Y1, X2, X8, and X9 for each provincial unit.

The key influencing factors of farmland transfer area were analyzed using geographic detector. Factor detection measures the explanatory power of various influencing factors on farmland transfer. Among them, the "regional farmland area" showed the strongest explanatory power on farmland transfer, with a q-value of 0.663, significantly surpassing other factors. The remaining factors demonstrated the following levels of explanatory power: "proportion of family farms supported by loans" (0.319) > "proportion of non-agricultural population" (0.225) > "grain yield per unit area" (0.211) > "GDP per capita" (0.156) > "proportion of disaster-affected area" (0.134) > "proportion of farmland transferred to family farms and cooperatives" (0.131) > "total power of agricultural machinery per hectare" (0.123) > "proportion of migrant labor force" (0.089) > "the proportion of farmland transferred by leasing (subcontracting) " (0.067). The above results show that: (1) The amount of regional farmland determines the amount of farmland available for transfer to a large extent. The regions with larger regional farmland areas generally demonstrate a corresponding increase in farmland transfer areas, as illustrated in Fig. 12. For instance, Heilongjiang, Henan, and Inner Mongolia—the top three provinces in China in terms of farmland transfer area—also rank highest in terms of their national-scale regional farmland areas. (2) Policy factors play a crucial guiding role in agricultural and rural development. Despite the relatively small coverage of loan support, with the "proportion of family farms supported by loans" ranging from 0.1 to 10.5%, it still holds significant explanatory power as the second most influential factor. This indicates that national financial support for new agricultural management entities plays a vital role in facilitating farmland transfer. The analysis of Fig. 12 reveals that among the provincial units, approximately one-third exhibit a proportion of family farms supported by loans below 1%. These provinces and municipalities include Inner Mongolia, Shaanxi, Guangxi, Shanghai, Shandong, Henan, Fujian, Jilin, Liaoning, Beijing, Guangdong, Hebei, and Hainan. Notably, the farmland transfer area in these regions amounts to 15.924 million hectares, which is equivalent to 42.3% of China's total farmland. Given the significant impact of "the proportion of family farms supported by loans" on farmland transfer areas, it is imperative to enhance financial subsidies for family farms. This is particularly crucial in Shandong Province, as well as Inner Mongolia and Henan, which have a combined farmland transfer area exceeding 2 million hectares but possess an alarmingly low proportion of family farms supported by loans. Therefore, these regions should be prioritized for preferential policies. (3) Urbanization plays a crucial role in facilitating the transfer of farmland, as indicated by the index value of the "proportion of non-agricultural population", which ranges from 50.1 to 89.3%. The higher the proportion of non-agricultural population, the weaker the connection between individuals and farmland becomes. The process of rural-to-urban migration represents a separation between production and living spaces from agricultural land. Farmland outflow typically occurs during this population movement. The data presented in Fig. 12 indicates that the proportion of non-agricultural population in Shanghai, Beijing, Tianjin, Guangdong, Jiangsu, Zhejiang, Liaoning, Chongqing, Fujian, Inner Mongolia, Heilongjiang, and Ningxia ranges from 64.5 to 89.3%, all surpassing the national average. This has effectively facilitated the process of transferring farmland. Conversely, Hunan, Anhui, Sichuan, and Henan, major grain-producing regions, have a proportion of non-agricultural population of less than 60%. Considering the substantial farmland resources in these areas, increasing the proportion of non-agricultural population would aid in further expanding the area available for farmland transfer. (4) The explanatory power of "grain yield per unit area" and "GDP per capita", which reflect the comprehensive capacity for grain production and regional economic development level, ranked fourth and fifth, respectively. The output capacity of farmland itself and the regional economic level had a positive impact on farmland transfer. (5) The q-values of other factors ranged from 0.046 to 0.168, indicating relatively weak explanatory power.

**Figure 13:**
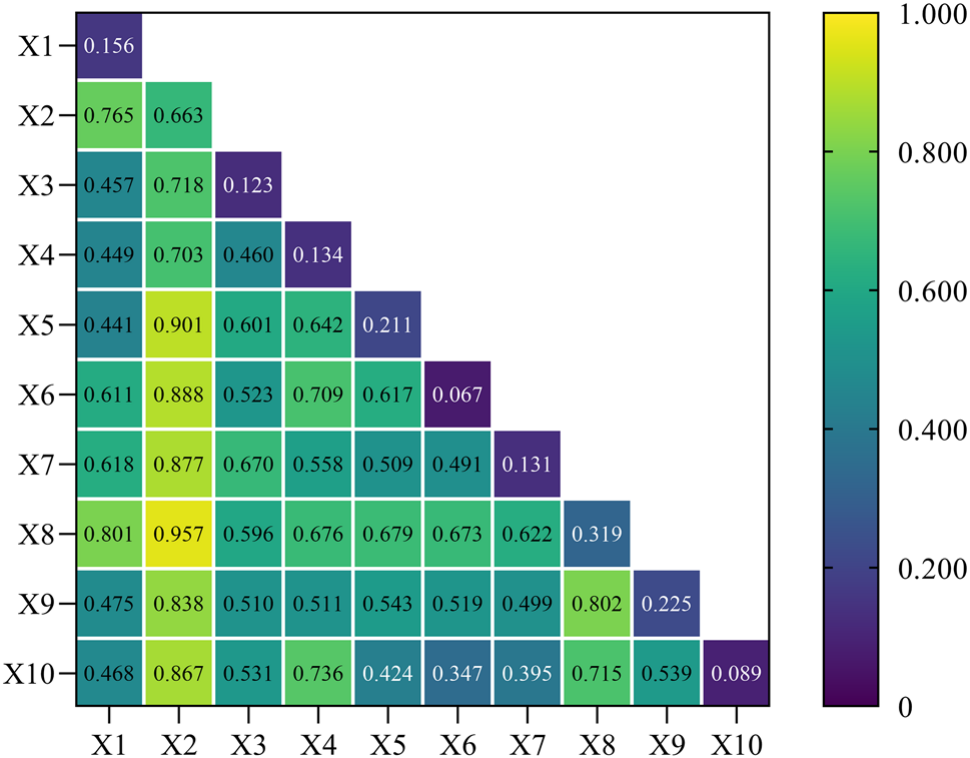
Q value of interactive detection at the national scale.

The interaction detection primarily examined whether there were any interaction effects among the 10 influencing factors on farmland transfer. The findings indicate that 40 groups exhibit nonlinear enhancement, specifically q(X1 ∩ X2) > (q(X1) + q(X2)), while 5 groups demonstrate double-factor enhancement, namely q(X1 ∩ X2) > Max(q(X1), q(X2)). These results suggest that the explanatory power of any two factors on the pattern of farmland transfer will be significantly enhanced through their interaction. The “regional farmland area” exhibits the highest explanatory power, as depicted in Fig. 13. Furthermore, its explanatory power is significantly enhanced through interaction with other factors. Notably, the interactive explanatory powers of "regional farmland area" with "family farm loan support ratio" and "grain production per unit area" are 0.957 and 0.901, respectively. Moreover, after interacting with factors, such as "the proportion of farmland transferred by leasing (subcontracting)", "proportion of farmland flowing into family farms and cooperatives", "proportion of migrant labor force", and "proportion of non-agricultural population", the explanatory power consistently exceeds 0.8. This implies that deploying these factors in regions abundant in total farmland resources would exert a substantial impact on farmland transfer dynamics. In addition, there are two groups of factors whose interaction yields an explanatory power greater than 0.8. Among them, the interaction between "GDP per capita" and "proportion of family farms supported by loans" has an explanatory power of 0.801, while the interaction between "proportion of family farms supported by loans" and "proportion of non-agricultural population" has an explanatory power of 0.802. This suggests that regional economic level and policy support, as well as policy support and urbanization level, can jointly exert a significant influence on the pattern of farmland transfer.

#### Key factors influencing farmland transfer rate

**Figure 14:**
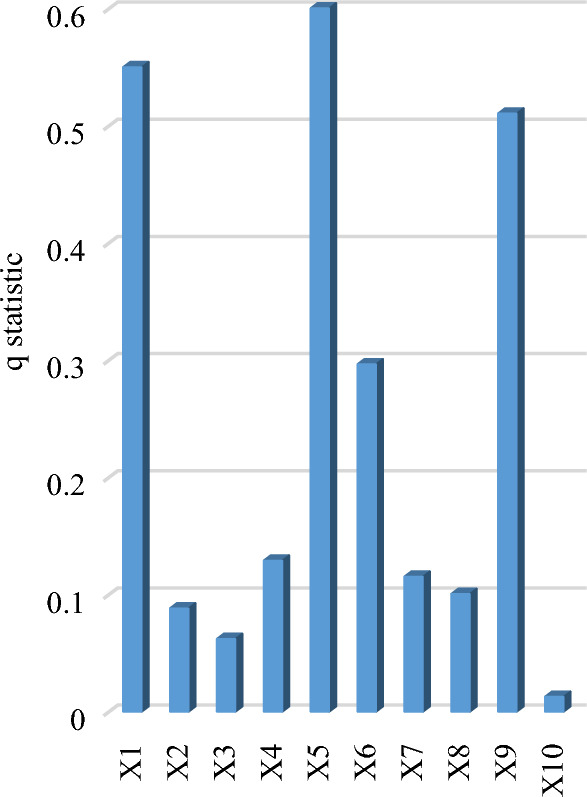
Factor analysis results of geographic detector.

**Figure 15:**
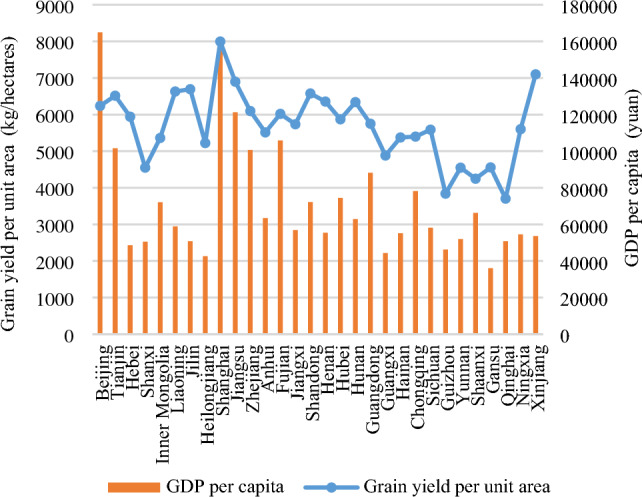
Comparative analysis of evaluation indicators X1 and X5 of each provincial unit.

The factors influencing the transfer rate of farmland differ from those impacting the area of farmland transfer. The factor analysis results presented in Fig. 14 reveal that "grain yield per unit area", "GDP per capita," and the "proportion of non-agricultural population" emerge as the primary determinants influencing the rate of farmland conversion. With explanatory power of 0.600, 0.550, and 0.511, respectively. It can be seen that the rate of farmland transfer is closely correlated with the regional output level of farmland, economic development level, and urbanization level. Enhancing the production capacity of farmland, developing non-agricultural industries, and increasing employment levels can effectively facilitate regional farmland transfer. As the indicators for the "proportion of non-agricultural population" in each province have been previously discussed, Fig. 15 only illustrates the regional disparities in "grain yield per unit area" and "GDP per capita" among the top three influencing factors. The figure reveals that more than half of the top 10 provinces in terms of "grain yield per unit area" and "GDP per capita" also rank among the top 10 in terms of their farmland conversion rate. Notably, Beijing, Tianjin, Shanghai, and Jiangsu exhibit exceptionally high levels of "grain yield per unit area", "GDP per capita", and "farmland transfer rate". Further analyzing the regions exhibiting low values of these two indicators, the bottom 10 provinces in terms of "grain yield per unit area" include Qinghai, Guizhou, Shanxi, Yunnan, Shanxi, Gansu, Guangxi, Heilongjiang, Inner Mongolia, and Hainan. Their grain yield per unit area ranges from 3703.4 to 5375.0 kg/ha. Among them, Heilongjiang and Inner Mongolia have a farmland transfer rate of 57.9% and 39.1%, respectively, while other regions have rates ranging between 9.2 and 26.6%, which are lower than the national average. The 10 provinces with the lowest "GDP per capita" index values include Gansu, Heilongjiang, Guangxi, Guizhou, Hebei, Shanxi, Jilin, Qinghai, Yunnan, and Xinjiang. Their GDP per capita ranges from 35,995 to 53,593 yuan. The farmland transfer rates in Heilongjiang and Jilin are recorded at 57.9% and 41.4% respectively. In other regions, the farmland transfer rate falls between 10.9 and 35.5%, which is below the national average level. It can be observed that areas with low grain yield per unit area and GDP per capita generally exhibit a lower farmland transfer rate. In addition, the influencing factors from the fourth to seventh positions are the "proportion of farmland transferred through leasing (subcontracting)", "proportion of disaster-affected area", "proportion of farmland transferred to family farms and cooperatives", and "proportion of family farms supported by loans". These factors have an explanatory power greater than 0.1 and are closely associated with agricultural production indicators. This indicates that improving the channels for farmland transfer, reducing crop disaster occurrences, fostering new agricultural management entities such as family farms and specialized cooperatives, and increasing financial support for large-scale operations can all contribute to promoting regional farmland transfer processes. It should be noted that the variable "regional farmland area" has minimal influence on farmland transfer, ranking eighth with an explanatory power of only 0.088. The results above indicate that the influence of regional farmland area on the rate of farmland transfer is negligible, suggesting that the quantity of farmland does not have significant impact on the regional farmland transfer rate.

**Figure 16:**
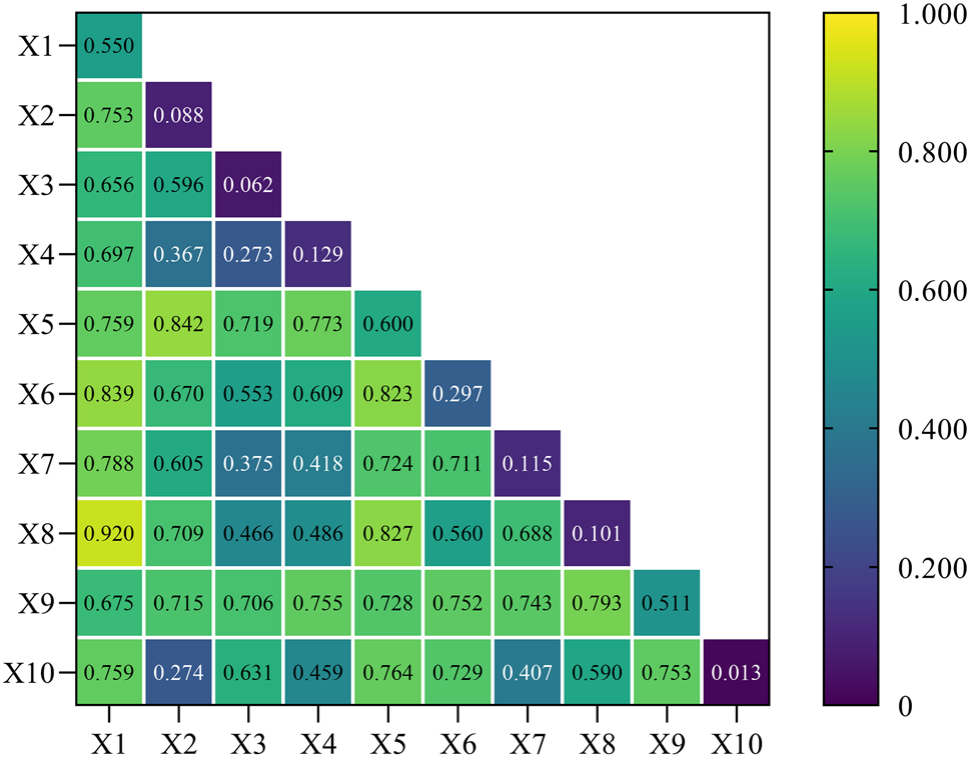
Q value of interactive detection at the provincial scale.

The 45 interaction relationships consisted of 36 nonlinear enhancements and 9 two-factor enhancements. Due to their robust explanatory power regarding the farmland transfer rate, "grain yield per unit area" and "GDP per capita" demonstrate strong interactive explanatory abilities with other factors. Specifically, the interactive explanatory power of "grain yield per unit area" with "regional farmland area," "proportion of family farms supported by loans," and "proportion of farmland under lease (subcontract) transfer" exceeded 0.8, reaching values of 0.842, 0.827, and 0.823, respectively. Similarly, the interactive explanatory power of "GDP per capita" with "the proportion of family farms supported by loans" and "the proportion of farmland transferred by leasing (subcontracting)" reached values of 0.920 and 0.839, respectively. These findings underscore the significance of national financial support and effective farmland transfer methods in improving farmland transfer rates in both typical agricultural areas and economically developed regions. Furthermore, when interacting with other indicators, both "grain yield per unit area" and "GDP per capita" exhibited an explanatory power exceeding 0.6. Similarly, various factors such as "regional farmland area", "proportion of non-agricultural population", and "proportion of migrant labor" also demonstrated an explanatory power exceeding this threshold when interacting with different variables. These significant interactions among index combinations can serve as valuable references for farmland transfers across diverse types of areas (Fig. 16).

## Conclusion and discussion

### Research findings

Firstly, the farmland transfer in China has entered a phase of sluggish growth, with the total amount increasing annually but at a decelerated rate. Currently, leasing (subcontracting) represents the predominant mode of farmland transfer, accounting for 84.1% of the transferred area, while other forms such as transfer, exchange, and share cooperation constitute 2.4%, 3.3%, and 5.2%, respectively. Provincial units primarily adopt the "leasing (subcontracting) + " model for farmland transfers. In 2020, farmers accounted for 60.1% of the recipients of transferred farmland, followed by specialized cooperatives (21.5%), enterprises (10.4%), and other entities (7.9%). The distribution pattern essentially establishes a composition of farmland transfer objects dominated by farmers and supplemented by specialized cooperatives.

Secondly, there are disparities in the key factors that influence the area and rate of farmland transfer. The area of farmland transferred is primarily influenced by the total regional farmland area. In terms of specific factors, the "regional farmland area," "proportion of family farms supported by loans," and "proportion of non-agricultural population" exert the most significant impact on the extent of farmland transfer. This demonstrates that the quantity of available farmland resources, the level of financial support intensity, and the degree of urbanization all play crucial roles in promoting an increase in transferred agricultural lands. On the other hand, the rate at which farmland is transferred is mainly affected by the level of agricultural output, regional economic development status, and urbanization rate. Higher levels of agricultural productivity stimulate voluntary expansion among agricultural producers and operators in terms of their operational scale. Urban development simultaneously increases its attractiveness to agricultural populations who are involved in non-agricultural industries, thereby directly contributing to a higher regional farmland transfer rate.

Thirdly, the explanatory power of the interaction between any two factors on farmland transfer is significantly enhanced. When analyzing the farmland transfer area as the dependent variable, "regional farmland area" emerges as the single factor with the highest explanatory power. The interactive explanatory powers of "proportion of family farms supported by loans" and "grain yield per unit area" are 0.957 and 0.901, respectively. Moreover, its interactive explanatory power, with indicators representing modes of farmland transfer, transfer objects, and non-agricultural employment, also exceeds 0.8. These research findings can provide scientific guidance for promoting the efficient utilization of farmland resources in regions abundant in land resources and accelerate China's overall farmland transfer process. In analyzing the farmland transfer rate as the dependent variable, it was observed that the interactive explanatory power between "grain yield per unit area", "GDP per capita", and other factors exceeded 0.6. Notably, the interactive explanatory power between the "proportion of family farms supported by loans" and the "proportion of farmland transferred by leasing (subcontracting)" surpassed 0.8. These research findings can serve as a valuable reference for promoting effective strategies in transferring agricultural land within typical agricultural regions or areas with high levels of economic development.

### Research recommendations

#### Limitations

This paper focuses on the temporal and spatial patterns of farmland transfer and its influencing factors after China's farmland transfer enters a period of slow growth, aiming to achieve the research objective. In future studies, we will strive to address the limitations of this paper. (1) Obtaining data on a longer time scale: While we can gather data on the extent of farmland transfers over an extended period, obtaining detailed classification data on the objects and modes involved in these transfers remains challenging. Therefore, obtaining more long-term data would contribute to a comprehensive and systematic understanding of past and future farmland transfers in China. (2) There is a need for a more comprehensive selection of indicators that reflect policy factors. In recent years, the Chinese government has implemented various agricultural policies, including direct grain subsidies, improved variety subsidies, price support policies, comprehensive agricultural material subsidies, and producer subsidies. Studies have shown that these policies can have both positive and negative effects on farmland conversion^36–39^. To assess the impact of agricultural policies in different regions, this study focuses on the "the proportion of family farms supported by loans" as an indicator because of the strong regional uniqueness of many agricultural policies. Future studies could explore the development of indicators tailored to specific agricultural policies implemented in various regions^39^. (3) Include indicators that reflect farmers' subjective preferences. While indicators reflecting farmers' subjective preferences are typically found in micro-level studies, it is important to acknowledge that the transfer of farmland at any scale represents the subjective decision-making of agricultural producers^40^. In future research, it may be appropriate to include an index that reflects farmers' willingness to transfer farmland.

### Policy implications

1. Where will China's farmland transfer move forward in the future? The findings indicate that the regional farmland area and GDP per capita are the primary influencing factors for both the area and rate of farmland conversion, with explanatory powers of 0.631 and 0.600, respectively. This suggests that there are two key types of areas suitable for China's farmland transfer^41,42^. These can be broadly categorized as "large agricultural areas" and "metropolitan suburbs". The former relies on agriculture as a traditional pillar industry, possesses abundant farmland resources, and has a high per capita share. These regions contribute to national food security while benefiting from favorable agricultural policies provided by the government. On the other hand, "metropolitan suburbs" not only have well-developed secondary or tertiary industries but also encompass districts and counties with advantageous agricultural production functions. Moreover, central cities or non-agricultural industries within these areas exhibit particularly strong absorption capacities for agricultural populations. ① Provinces with a relatively high area of farmland circulation are typical representatives of "large agricultural areas," including Heilongjiang, Shandong, Anhui, Inner Mongolia, and Henan. These provinces account for 31.8% and 39.7% of the total farmland area and farmland transfer area in China, respectively. Despite being influenced by recent changes in grain purchase policies and subsidy policies, the process of farmland transfer has shown a slowing trend. Nevertheless, it remains the primary region for future farmland transfers. The secondary regions mainly include Jiangsu, Jilin, Sichuan, Hebei, Hunan, Hubei, and Liaoning, which are key grain-producing areas in China. These regions account for 29.5% and 32.1% of the total farmland area and farmland transfer area in China, respectively. The range of farmland transfer areas in each province generally falls between 1.2 million to 2.2 million hectares, which is approximately one-third of their total farmlands. This indicates significant potential for further circulation^43^. ② Typical big cities such as Beijing, Tianjin, and Shanghai are not the primary regions for cultivating the "metropolitan suburbs" type. Despite their high per capita GDP, urbanization rate, and farmland transfer rate, these regions lack a strong agricultural production foundation on one hand and have limited potential for further promoting farmland transfer on the other hand. The development of "metropolitan suburbs" is mainly concentrated in Jiangsu and Zhejiang provinces because of their advanced secondary and tertiary industry development, per capita GDP exceeding 100,000 yuan, and an urbanization rate surpassing 70%. These areas were traditionally agricultural regions where some agricultural districts and counties have been preserved. The economic development in such areas can effectively leverage the attraction of rural labor force while facilitating the transfer of cultivated land. If expansion is considered, Guangdong, Fujian, Chongqing, and other regions could be listed as potential targets for cultivating the " metropolitan suburbs" type.
2. How to promote the transfer of farmland in different types of areas in the future. "Large agricultural areas" and "metropolitan suburbs" are identified as key regions for promoting farmland transfer in China, while other areas can be temporarily classified as secondary regions. The appropriate methods for each type of region vary accordingly. ① The focus should be on "policy support + yield increase" when transferring farmland in "large agricultural areas". The regional area of farmland is the most significant factor influencing the transfer area, with a high correlation coefficient of 0.957 with the proportion of family farms supported by loans and grain yield per unit area, which has a correlation coefficient of 0.901. Enhancing policy support for regions abundant in farmland resources and comprehensively improving seed quality, technology application, and high-standard farmland construction will provide a stable driving force for subsequent farmland transfers^44,45^. For instance, provinces such as Heilongjiang, Liaoning, Henan, Inner Mongolia, Jilin, Sichuan, Shandong, and Hebei, which have relatively high rates (> 30%) of farmland transfer, should receive increased subsidies. It is essential to strictly control against non-agricultural or non-grain conversions during this process to preserve the agricultural nature of these lands, especially for food cultivation^46^. ② The metropolitan suburbs should leverage the driving force of the secondary and tertiary industries in developed regions. They should prioritize the cultivation of inflow objects such as family farms and professional cooperatives on farmland, streamline the circulation channels for farmland, standardize circulation practices, and safeguard the rights and interests of all parties involved. For instance, Zhejiang and Jiangsu provinces should strive to retain and extensively explore districts and counties with agricultural production attributes. They should foster family farm models and specialized cooperative models with distinctive Chinese characteristics during the transfer of farmland. Gradually enhancing regional agricultural production functions with unique features, they should aim for self-sufficiency in grain production while reducing excessive reliance on major grain-producing areas. This will serve the overall national food security situation. ③ The transfer of farmland in the third type area should explore an appropriate approach based on the original regional advantages. For example, in Beijing, Shanghai, and Tianjin, where the farmland transfer rate is already high but not mainly for agricultural production, it is inappropriate to further increase the transfer rate as a goal. Instead, it is more crucial to enhance agricultural and rural land policies and prohibit non-agricultural use of transferred farmland. In the grain production and marketing balance areas of central and western China, a targeted approach should be adopted. This approach should consider key factors and regional advantages of farmland transfer to enhance the regional economic level, urbanization level, and farmland output^47^. By strengthening specific core indicators with robust interactive explanatory power, the linkage effect of farmland transfer can be promoted^48^.

### Supplementary Information

Supplementary Information 1.

Supplementary Information 2.

Supplementary Information 3.

Supplementary Information 4.

Supplementary Information 5.

## Data Availability

All data generated or analysed during this study are included in this published article and its Supplementary Information files.
